# Supplementary material for: Movement and habitat selection of the western spadefoot (Spea hammondii) in southern California
Source: PLoS One. 2019 Oct 3;14(10):e0222532. doi: 10.1371/journal.pone.0222532 (PMC6776459; doi:10.1371/journal.pone.0222532)
Supplement: Attachment — Submitted filename: PONE-D-19-16369_reviewer.pdf [file pone.0222532.s002.pdf]

PLOS ONE

Movement and habitat selection of the western spadefoot (Spea hammondii) in southern California

--Manuscript Draft--

**Manuscript Number:**	PONE-D-19-16369
**Article Type:**	Research Article
**Full Title:**	Movement and habitat selection of the western spadefoot (Spea hammondii) in southern California
**Short Title:**	Movement and habitat selection of the western spadefoot (Spea hammondii) in southern California
**Corresponding Author:**	Katherine Baumberger<br>US Geological Survey Western Ecological Research Center<br>Santa Ana, California UNITED STATES
**Keywords:**	California; dispersal; fossorial anuran; minimum convex polygon; radio telemetry; rainfall; site fidelity; soil texture; Spea hammondii; upland habitat; spadefoot; local convex hull
**Abstract:**	Agricultural activity, urban development and habitat alteration have caused the disappearance of the western spadefoot (Spea hammondii) from 80% of its geographic range in southern California. Despite the western spadefoot's continuing decline, little research has been conducted on its natural history. The home range of adult spadefoots is unknown, and their use of upland habitat is poorly understood. Both factors are important for the long-term conservation of the species because adult spadefoots spend the majority of their lives away from breeding pools in self-excavated burrows. Between January 2012 and January 2013, we surgically implanted radio transmitters in 15 spadefoots at two locations and recorded their movements and habitat use. The mean distance moved between burrow locations was 18 m (SD ± 24.1 m, min = 1 m - max = 204 m). The mean distance of burrows from the breeding pools was 40 m (SD ± 37.42, min = 1 m – max = 262 m). Rain was a significant predictor of spadefoot movement, with more rain predicting higher probability of movement and larger distances moved. At remote sensing (1 m) scale spadefoots selected grassland habitat for their burrow locations. At the microsite scale (< 1 m) spadefoots strongly selected duff over grass or shrub cover. Spadefoots burrowed in friable, sandy/loam soil with significantly less clay than random pseudoabsence points. This research enhances our understanding of a little-studied species and will contribute to the development of effective management plans for the western spadefoot.
**Order of Authors:**	Katherine Baumberger
	M.V. Eitzel
	Matthew E. Kirby
	Michael H. Horn
**Additional Information:**	
**Question**	**Response**
**Financial Disclosure**<br><br>Enter a financial disclosure statement that describes the sources of funding for the work included in this submission. Review the submission guidelines for detailed requirements. View published research articles from *PLOS ONE* for specific examples.	K. B. received funding from the Laguna Canyon Foundation (https://lagunacanyon.org/), the Association of Zoos and Aquariums Amphibian Taxon Advisory Group Conservation Fund (https://www.aza.org/amphibian-conservation), and the Natural Communities Coalition (formally the Nature Reserve of Orange County) (https://occonservation.org/). The funders had no role in study design, data collection and analysis, decision to publish, or preparation of the manuscript.

Powered by Editorial Manager® and ProduXion Manager® from Aries Systems Corporation

This statement is required for submission and **will appear in the published article** if the submission is accepted. Please make sure it is accurate.<br><br>**Unfunded studies**<br>Enter: *The author(s) received no specific funding for this work.*<br><br>**Funded studies**<br>Enter a statement with the following details:<br>• Initials of the authors who received each award<br>• Grant numbers awarded to each author<br>• The full name of each funder<br>• URL of each funder website<br>• Did the sponsors or funders play any role in the study design, data collection and analysis, decision to publish, or preparation of the manuscript?<br>• **NO** - Include this sentence at the end of your statement: *The funders had no role in study design, data collection and analysis, decision to publish, or preparation of the manuscript.*<br>• **YES** - Specify the role(s) played.<br><br>* typeset	
**Competing Interests**<br><br>Use the instructions below to enter a competing interest statement for this submission. On behalf of all authors, disclose any competing interests that could be perceived to bias this work—acknowledging all financial support and any other relevant financial or non-financial competing interests.<br><br>This statement **will appear in the published article** if the submission is accepted. Please make sure it is accurate. View published research articles from *PLOS ONE* for specific examples.	The authors have declared that no competing interests exist.

**NO authors have competing interests**<br><br>Enter: *The authors have declared that no competing interests exist.*<br><br>**Authors with competing interests**<br><br>Enter competing interest details beginning with this statement:<br><br>*I have read the journal's policy and the authors of this manuscript have the following competing interests: [insert competing interests here]*<br><br>* typeset	
**Ethics Statement**<br><br>Enter an ethics statement for this submission. This statement is required if the study involved:<br><br>• Human participants<br>• Human specimens or tissue<br>• Vertebrate animals or cephalopods<br>• Vertebrate embryos or tissues<br>• Field research<br><br>Write "N/A" if the submission does not require an ethics statement.<br><br>General guidance is provided below. Consult the submission guidelines for detailed instructions. **Make sure that all information entered here is included in the Methods section of the manuscript.**	This study was conducted under California Department of Fish and Wildlife permit SC-07437 and approved by the Institutional Animal Care and Use Committee at California State University, Fullerton (Protocol No. 11-R-05). Animal handling followed the Herpetological Animal Care and Use Committee of the American Society of Ichthyologists and Herpetologists guidelines. Animals were anaesthetized via immersion in MS-222 (0.4g dissolved in 500ml of water) at a veterinary clinic, and every effort was made to minimize suffering.

**Format for specific study types**<br><br>**Human Subject Research (involving human participants and/or tissue)**<br>• Give the name of the institutional review board or ethics committee that approved the study<br>• Include the approval number and/or a statement indicating approval of this research<br>• Indicate the form of consent obtained (written/oral) or the reason that consent was not obtained (e.g. the data were analyzed anonymously)<br><br>**Animal Research (involving vertebrate animals, embryos or tissues)**<br>• Provide the name of the Institutional Animal Care and Use Committee (IACUC) or other relevant ethics board that reviewed the study protocol, and indicate whether they approved this research or granted a formal waiver of ethical approval<br>• Include an approval number if one was obtained<br>• If the study involved *non-human primates*, add *additional details* about animal welfare and steps taken to ameliorate suffering<br>• If anesthesia, euthanasia, or any kind of animal sacrifice is part of the study, include briefly which substances and/or methods were applied<br><br>**Field Research**<br><br>Include the following details if this study involves the collection of plant, animal, or other materials from a natural setting:<br>• Field permit number<br>• Name of the institution or relevant body that granted permission	
**Data Availability**<br><br>Authors are required to make all data underlying the findings described fully available, without restriction, and from the time of publication. PLOS allows rare exceptions to address legal and ethical concerns. See the PLOS Data Policy and FAQ for detailed information.	Yes - all data are fully available without restriction

A Data Availability Statement describing where the data can be found is required at submission. Your answers to this question constitute the Data Availability Statement and **will be published in the article**, if accepted.<br><br>**Important:** Stating 'data available on request from the author' is not sufficient. If your data are only available upon request, select 'No' for the first question and explain your exceptional situation in the text box.<br><br>Do the authors confirm that all data underlying the findings described in their manuscript are fully available without restriction?	
**Describe where the data may be found in full sentences. If you are copying our sample text, replace any instances of XXX with the appropriate details.**<br><br>• If the data are **held or will be held in a public repository**, include URLs, accession numbers or DOIs. If this information will only be available after acceptance, indicate this by ticking the box below. For example: *All XXX files are available from the XXX database (accession number(s) XXX, XXX.*).<br>• If the data are all contained **within the manuscript and/or Supporting Information files**, enter the following: *All relevant data are within the manuscript and its Supporting Information files.*<br>• If neither of these applies but you are able to provide **details of access elsewhere**, with or without limitations, please do so. For example:<br><br>*Data cannot be shared publicly because of [XXX]. Data are available from the XXX Institutional Data Access / Ethics Committee (contact via XXX) for researchers who meet the criteria for access to confidential data.*<br><br>*The data underlying the results presented in the study are available from (include the name of the third party*	All data files are available from the Dryad database (doi:10.5061/dryad.8359820, https://datadryad.org/review?doi=doi:10.5061/dryad.8359820).

and contact information or URL).<br>• This text is appropriate if the data are owned by a third party and authors do not have permission to share the data.<br><br>* typeset	
Additional data availability information:	

Manuscript
Click here to download Manuscript Baumberger_etal_WesternSpadefootMs_9June2019.doc

Movement and habitat selection of the western spadefoot (*Spea hammondii*) in southern California

Katherine L. Baumberger[1*,#a], M. V. Eitzel[2,#b], Matthew E. Kirby[3], and Michael H. Horn[4]

[1] Environmental Studies Program, California State University at Fullerton, Fullerton, California, USA

[2] Department of Environmental Science, Policy, and Management, University of California at Berkeley, Berkeley, California, USA

[3] Department of Geological Sciences, California State University at Fullerton, Fullerton, California, USA

[4] Department of Biological Science, California State University at Fullerton, Fullerton, California, USA

[#a] Current address: Western Ecological Research Center, United States Geological Survey, Santa Ana, California, USA

[#b] Current address: Science and Justice Research Center, University of California at Santa Cruz, Santa Cruz, California, USA

* Corresponding author

E-mail: kbaumberger@usgs.gov (KLB)

Abstract

Agricultural activity, urban development and habitat alteration have caused the disappearance of the western spadefoot (*Spea hammondii*) from 80% of its geographic range in southern California. Despite the western spadefoot's continuing decline, little research has been conducted on its natural history. The home range of adult spadefoots is unknown, and their use of upland habitat is poorly understood. Both factors are important for the long-term conservation of the species because adult spadefoots spend the majority of their lives away from breeding pools in self-excavated burrows. Between January 2012 and January 2013, we surgically implanted radio transmitters in 15 spadefoots at two locations and recorded their movements and habitat use. The mean distance moved between burrow locations was 18 m (SD ± 24.1 m, min = 1 m - max = 204 m). The mean distance of burrows from the breeding pools was 40 m (SD ± 37.42, min = 1 m – max = 262 m). Rain was a significant predictor of spadefoot movement, with more rain predicting higher probability of movement and larger distances moved. At remote sensing (1 m) scale spadefoots selected grassland habitat for their burrow locations. At the microsite scale (< 1 m) spadefoots strongly selected duff over grass or shrub cover. Spadefoots burrowed in friable, sandy/loam soil with significantly less clay than random pseudoabsence points. This research enhances our understanding of a little-studied species and will contribute to the development of effective management plans for the western spadefoot.

Introduction

Habitat loss is one of the main causes of amphibian decline throughout the world [1-4]. Because of this loss, amphibian conservation has historically been focused on the preservation or restoration of aquatic habitats [5], and on the upland habitat use of adult amphibians [6-7]. Specifically, attention has been given to determining the minimum buffer around a breeding pool required to conserve the adult population [8-11]. Semlitsch and Brodie [12] found the mean core terrestrial habitat for amphibians to be 205—368 m from the edge of aquatic habitat, while the California Department of Fish and Wildlife [13] requires a 76 m buffer around known vernal pools to preserve upland habitat. Determining adequate terrestrial buffers is key to ensuring the viability of an amphibian population, and one buffer size may not fit all amphibians [5].

For many of California’s amphibian species very little is known regarding their terrestrial habitat use, including for 19 of the pond-breeding Species of Special Concern [14]. These amphibians may not be detected when an environmental impact assessment is done for a proposed change in land use, and therefore habitat area and specific habitat requirements can be underestimated. Thus, there is a practical need to characterize home range and habitat selection of these pond-breeding species.

We examined use of upland habitat by the western spadefoot (*Spea hammondii*), a burrowing anuran that has been extirpated from 80% of its range in southern California because of agricultural expansion and urban development [15-16]. This extensive habitat loss has led to the western spadefoot’s listing as a California Species of Special Concern [14], and the species in under review for listing as endangered or threatened under the Endangered Species Act [17]. The western spadefoot is endemic to California and historically inhabited lowlands such as river floodplains and washes in the Central Valley of California, and along the coast from central California to northwestern Baja California [16,18]. Adult spadefoots spend most of their time

underground and emerge primarily on rainy nights to feed and to breed in ephemeral pools [19-20]. The home range of adult western spadefoots is unknown, and the animal's use of terrestrial habitat is poorly understood [14,21].

The objective of this baseline study was to determine the movement ecology and microhabitat use of spadefoots at two sites in Orange County, California. We used radio-telemetry to monitor 15 spadefoots to establish a baseline understanding of four aspects of their habitat use patterns: 1) basic movement ecology, including home range size and distances moved away from breeding pools; 2) identification of variables predicting movement; 3) vegetation characteristics at burrow locations (the 1 x 1 m area encompassing the spadefoot's position); and 4) soil characteristics of aestivation locations (defined as a residence exceeding three weeks in the same burrow location). We were thus able to contribute to basic understanding of the movement and habitat selection of the western spadefoot which can underlie future studies and management planning.

Materials and Methods

Ethics statement

This study was conducted under California Department of Fish and Wildlife permit SC-07437 and approved by the Institutional Animal Care and Use Committee at California State University, Fullerton (Protocol No. 11-R-05). Animal handling followed the Herpetological Animal Care and Use Committee of the American Society of Ichthyologists and Herpetologists guidelines [22].

Animals were anaesthetized via immersion in MS-222 (0.4g dissolved in 500ml of water) at a veterinary clinic, and every effort was made to minimize suffering.

Study Area

This study was conducted in two protected parks, Crystal Cove State Park and the Laguna Coast Wilderness Park in Orange County, California. Together, these two areas form the largest remaining contiguous parcels of coastal sage scrub habitat in Orange County. These sites are also home to some of the few remaining vernal pools in the area. The site at Crystal Cove State Park is in a campground with greater potential for human nighttime activity. The site in the Laguna Coast Wilderness is a short drainage ditch along a popular hiking trail. Both areas were closed for several days following rain events to prevent visitors from damaging the wet trails. These closures reduced the impact of human interference on spadefoot movement by limiting the usual heavy human traffic at the study sites. All necessary permits were obtained from both Crystal Cove State Park and Orange County Parks, and our study complied with all relevant regulations.

The area of the Crystal Cove State Park breeding pool was approximately 38 m^2, while the Laguna Coast Wilderness pool was 3 m^2. However, during the 2011-2012 breeding season the Crystal Cove pool did not hold water. The Laguna Coast pool only held water for a week. The total precipitation during our study period (20.4 cm) was well below the 60-year average for the area (mean = 32.7 cm, SD ± 17.1). The average temperature for January through December 2012 (mean = 16.7° C, SD ± 5.5) was similar to the 60-year average (mean = 16.2° C, SD ± 1.29) [23].

Radio Telemetry

During the potential breeding period, between the end of January and the end of April 2012, we opportunistically captured a total of 15 spadefoots (see S1 Table for capture dates). Seven of the 15 animals were caught in Crystal Cove State Park and eight in the Laguna Coast Wilderness. Only three female spadefoots were captured, all at Crystal Cove State Park. We surgically implanted small radio transmitters (Model A2455, Advanced Telemetry Systems, Isanti, MN) into the coelomic cavities of each animal using the methods of Timm et al. [24]. We excavated the spadefoots from their burrows one time during the study to perform a welfare check approximately a week after their surgeries and no ill effects were observed.

We tracked the animals with a three-element Yagi antenna and a portable receiver (model TR-4, Telonics, Mesa, AZ) two times a week from January to June 2012. From July through October 2012 we monitored the animal’s aestivation location every other week. From October 2012 through January 2013 we monitored the animals once a week until the batteries gave out on the transmitters. We recorded burrow locations with a hand-held GPS receiver (model Rino 520, Garmin International, Inc., Olathe, KS) and then uploaded the points to a Geographic Information System (GIS) (ArcGIS 10 and 10.1, ESRI, Redlands, CA).

Vegetation Characteristics

To document vegetation characteristics at burrow locations, we placed a 1 m x 1 m Polyvinyl Chloride (PVC) square centered at the burrow opening. Within the square, we visually determined percent cover of five vegetation classes: grass, forbs, shrubs, leaf litter (recently fallen leaves), and duff (dead and decomposing vegetation from previous seasons) [25] to the

nearest 5 percent. We also recorded topographic slope and aspect to the nearest degree with a hand-held compass (Brunton, Riverton, WY). See Table 1 for a summary of these variables. In October 2012, we took the same measurements at 102 random pseudoabsence locations generated with ArcGIS and Geospatial Modeling Environment 0.7.2.1 (GME) [26]. The points were within a 300 m radius of the breeding pool (the maximum single movement of our tracked spadefoots), and at least 1 m away from a known spadefoot burrow [27].Though there is some temporal mismatch between these pseudoabsence points and the known spadefoot points, we carefully distinguished between dead grasses and forbs from 2012 and duff from 2011 or earlier. The points were stratified proportionally between the two habitat types present at our sites, "grassland" and "shrub," as classified from heads-up digitization of a sub-meter spatial resolution satellite image (ESRI base map, sources: ESRI, DigitalGlobe, GeoEye, i-cubed, USDA, USGS, AEX, Getmapping, Aerogrid, IGN, IGP, swisstopo, and the GIS User Community). We chose to stratify proportionally between these two habitat types because we did not have previous knowledge regarding which type the spadefoots would select and wanted to ensure adequate representation of both habitat types.

Table 1. Summary of habitat and rainfall characteristics

	**Mean**	**SD**	**Range**
**Percent sand**	52	15	29 – 82
**Percent silt**	38	11	13 – 53
**Percent clay**	10	4.6	3.8 – 18
**Total organic matter (percent)**	4.6	1.2	2.4 – 7.1
**Topographic slope (percent)**	6	6.5	0 – 30
**Aspect: northness**	-0.22	0.72	-1.0 – 1.0
**Aspect: eastness**	0.02	0.66	-1.0 – 1.0
**Percent grass cover**	25	28	0 – 100
**Percent shrub cover**	24	35	0 – 100
**Percent leaf litter cover**	15	33	0 – 100
**Percent forb cover**	15	20	0 – 100
**Percent duff cover**	40	36	0 – 100
**Duff height (cm)**	2.5	3	0 – 28
**Vegetation height (cm)**	45	36	0 – 150
**Locations with other animal burrows**	72/167	NA	NA
**Number of days with rainfall**	29	NA	NA
**Amount of rainfall (cm)**	0.41	0.89	0 – 4.5

Soil Characteristics

We followed the methods of Kirby et al. [28] to measure the percent total organic matter, percent silt, percent clay and percent sand of aestivation locations and pseudoabscence points. We used a core sampler to collect soil from 11 spadefoot burrows (several spadefoots aestivated within 1 m of each other) and 10 random points stratified spatially from our list of random points described above. The cores averaged 21.43 cm in length (range = 15 – 29 cm). This depth was consistent with the depth of burrows observed during welfare checks of the tracked animals (see Table [illegible]). Soil cores were subsampled every 2 cm for compositional analysis. These analyses included determining grain size and using the loss-on-ignition method to obtain percent total organic matter [29]. Grain-size was measured on a Malvern Mastersizer 2000 laser-diffraction grain-size analyzer coupled to a Hydro 2000G. All data were reported as percent by volume.

Grain-size data were classified using Wentworth's [30] classification, dividing clay at < 3.9 µm, silt at 3.9 – 62.5 µm, and sand at 62.5 – 2000 µm [31]. We averaged soil characteristics for the entire length of each core because spadefoots presumably moved through the entire profile to reach final placement in their burrows.

Data Analysis

We used ArcGIS and GME to estimate home range as a 95% Minimum Convex Polygon (MCP) for each site. We pooled data by site because of the low number of locations observed. We estimated the utilization distribution (UD) via the a-local convex hull (a-LoCoH) method [32-33] using the rhr package [34] in R [35]. The outputs from the UD analysis capture use of space at each site, rather than individual home ranges [33].

We used R for all the following statistical models. In all cases, we calculated p-values using likelihood ratio (LR) tests, comparing a full model including all variables with a reduced model eliminating the variable of interest. LR tests are more conservative than t-tests and are less sensitive to unbalanced designs [36]. Where models included only fixed effects, we used "glm" to fit models for LR tests and to obtain parameter estimates. Where models included a random effect (movement models), we used glmmADMB [37] to fit the generalized linear mixed models.

To evaluate the spadefoots' selection of burrowing location characteristics, we fit four separate binomial generalized linear models for spadefoot presence or pseudoabsence as predicted by: 1) each of the five vegetation classes, 2) site, vegetation height, duff depth, 3) slope and aspect (transformed into 'northness' = cos (aspect) and 'eastness' = sin (aspect)), and 4) the effect of other animal burrows (e.g. gopher and ground squirrel) on spadefoot presence. The models for these sets of burrow location characteristics were separate because the sample

sizes did not match (sample sizes for each set of models are listed in Results tables). Note that we did test the study site (Laguna Coast Wilderness versus Crystal Cove State Park) in these models, but as it was never significant it was not included in the results table. This outcome is unsurprising as we designed the study for equal numbers of toads at each site.

To evaluate the spadefoot's soil preferences at aestivation locations, we used binomial generalized linear models for spadefoot presence or pseudoabsence predicted by soil characteristics: soil texture (percent sand, silt, and clay) and total organic matter. Percent sand, silt, and clay are complementary (though in our measurements they do not always add to 100) and so we used a principal components analysis to combine them (using the “prcomp” function in R). The first principal component reflects the average soil texture of our sites, while the second component reflects the variation relative to that average. The third component reflects the degree to which the three textures do not sum to 100 percent, i.e. the analysis error. As this component accounted for very little variation, we did not include it in the binomial model for spadefoot presence/pseudoabsence. We created a similar generalized linear model for spadefoot presence or pseudoabsence predicted by total organic matter. Organic matter and soil texture variables were tested separately because of differing sample sizes

To determine drivers of spadefoot movement, we used a “hurdle” model [38] in which we first modeled the probability of movement (binomial generalized linear model) and then separately modeled the distances moved (gamma generalized linear model with a log link). Predictors in both models included rainfall in centimeters [39], sex of the spadefoot, phase of the moon [40], and study site (all as fixed effects), and individual spadefoot as a random effect. These models were fit using glmmADMB. Note that the sampling effort at the two sites was not equal, as the spadefoots were tagged much earlier at Laguna Coast Wilderness and therefore the

number of possible movements was higher. This difference in sampling effort was less important for the habitat models, but for the movement models it was critical that we control for site.

Results

The transmitters lasted an average of 272 days (ranged from 224 to 335 days). The maximum distance the spadefoots were found from the pools ranged from 16 to 262 m (Table 2, S1 Table), with a mean maximum distance of 69 m ± 61.48. The spadefoots used a mean of 13 burrows (SD ± 8.5), and the mean distance between burrow locations was 18 m (SD ± 24.2). They used 4-31 unique burrow sites (mean 11 ± 7.8) during the study. Nine of the 15 spadefoots (60%) reused one or more burrows at least once after moving to a different burrow. Outside of their aestivation period the spadefoots shifted their burrow location an average of every 8 ± 7 days, and 147/194 (~76%) movements between burrows were ≤ 25 m. Spadefoots began aestivating in May and June 2012. The spadefoots remained in their aestivation burrows 125 – 220 days (mean = 157 days ± 24.5) with the last spadefoot moving to a new burrow from its aestivation site on December 21, 2012. The 95% MCP home range size for Laguna Coast Wilderness was 8,242 m^2, larger than for Crystal Cove State Park which had a 95% MCP size of 6,285 m^2. The UD for the Laguna Coast Wilderness was also larger with an area of 1115 m^2. The UD for Crystal Cove was 599 m^2, roughly half (53%) of the UD of Laguna Coast Wilderness (Fig. 1). All the spadefoots were presumed to be alive at the end of the study, based on movement before transmitter failure. We observed no predation events or injuries. We did not remove the transmitters because of the potential negative consequences of removing transmitters that had been encapsulated by connective tissue [41].

Fig 1. Home range and utilization distribution of spadefoots.

Home ranges (black outline) represented as 95% minimum convex polygons, and utilization distribution (yellow polygons) of adult western spadefoots ~~(*Spea hammondii*)~~ in Orange County, California, USA, in 2012 for Laguna Coast Wilderness (left) and Crystal Cove State Park (right). Blue solid polygons show the breeding pool at each site.

Table 2. Summary of spadefoot characteristics and movement.

	Mean	SD	Range
**Snout-vent length (mm)**	62	5	54 – 70
**Mass (g)**	32	5	26 – 40
**Capture date**	2012/03/20	39 days	2012/01/21 – 2012/04/26
**Last located date**	2012/12/18	32 days	2012/10/13 – 2013/01/21
**Number of locations**	36	14	22 – 59
**Number of burrows used**	13	9	4 – 35
**Maximum distance from pool (m)**	69	60	16 – 262
**Home range size (MCP m^2)**	1340	1690	25 – 5620
**Mean distance between burrows (m)**	18	12	9 – 57
**Depth of burrow from welfare check (m)**	0.1	0.05	0.01-0.18

Drivers of Movement

Although spadefoots did move when no rain was present (Fig. 2), rain significantly predicted spadefoot movement, as did the animal random effect (Table 3). For an average animal, the model predicts a 46 percent chance the spadefoot will move with no rainfall (not significantly different than 50 percent), while at maximum rainfall (4.5 cm) the model predicts an 87 percent chance that the animal will move. We also found significantly more movement at Laguna Coast Wilderness, consistent with that site's longer sampling period. Number of meters moved was

predicted by rain in cm and by the animal random effect. With no rainfall, the model predicts that the average animal will move 21 m, while at maximum rainfall the model predicts that the average animal will move 163 m.

Fig 2. Spadefoot movement away from breeding pools vs. rainfall.

Spadefoot movement away from breeding pools over the course of the study (January 2012 through January 2013) at A) Laguna Coast Wilderness and B) Crystal Cove State Park. Daily rainfall in Orange County, USA, is displayed in gray bars in the background, and each line represents an individual spadefoot's distance from the breeding pool throughout the year. Most individuals remained within 100 m radial distance from the breeding pool, while one individual went much farther. Movements occurred outside rainfall events, and movement did not always occur during rainfall events, but statistically the relationship is significant (see Table 3). Individuals were tracked for a shorter time period at Crystal Cove State Park.

Table 3. Model untransformed parameter estimates, standard errors, and statistical significance.

Model	N	Parameter	Estimate	SE[1]	P-value
**Burrows: vegetation structure**	271	Vegetation height	NS	NS	NS
		Duff height	NS	NS	NS
**Burrows: vegetation cover**	300	Percent duff	0.01	0.005	0.001
		Percent shrubs	-0.02	0.006	0.002
		Percent forbs	NS	NS	NS
		Percent grass	-0.02	0.005	<0.001
		Percent leaf litter	NS	NS	NS
**Burrows: physiography**	285	Slope	-0.2	0.03	<0.001
		Aspect: northness	-0.55	0.22	<0.001
		Aspect: eastness	0.79	0.24	0.01
**Burrows: other burrow**	167	In other animal burrows	1.42	0.35	<0.001
**Aestivation: soil texture**	20	Principal component 1	NS	NS	NS
		Principal component 2	-2.79	1.55	0.03
**Aestivation: organic matter**	19	Total organic matter	NS	NS	NS
**Movement: yes/no**	532	Rainfall (cm)	0.46	0.11	< 0.001
		Mean site difference	-0.81	0.35	0.02
		Phase of moon	NS	NS	NS
		Sex of spadefoot	NS	NS	NS
		Animal random effect	0.33	NA	0.03
**Movement: distance (m)**	181	Rainfall (cm)	0.13	0.06	0.02
		Mean site difference	NS	NS	NS
		Phase of moon	NS	NS	NS
		Sex of spadefoot	NS	NS	NS
		Animal random effect	0.41	NA	< 0.001

[1]Standard errors for random effect variance components are not symmetrical and not included.

Characteristics of Burrow Locations

Duff depth and vegetation height were not significant predictors of spadefoot presence (Table 3). At the site scale (from the imagery-classified vegetation types), spadefoots strongly selected for grassland rather than shrubs: 140 of the 196 spadefoot locations were in grassland (71 percent), as compared to 14 percent grassland cover for all sites (Fig 3F). At the microsite scale, spadefoot strongly selected duff over grass or shrub cover (Figs 3A-E): the model predicts

spadefoot to choose a 100 percent grass site 32 percent of the time (significantly less than 50 percent), a 100 percent shrub site 42 percent of the time (significantly less than 50 percent), and a 100 percent duff site 95 percent of the time (significantly more than 50 percent). No other cover types were significant including bare ground (Table 3).

Fig 3. **Habitat characteristics of spadefoot burrow locations.**

Habitat characteristics within 1 m^2 of spadefoot burrow locations (P) and pseudoabsence (PA) locations in Orange County, California, USA, in 2012. Percent cover of A) duff, B) grass, C) shrubs, D) leaf litter, and E) forbs, in addition to F) the classification from high spatial resolution imagery of each presence or pseudoabsence point as "grass" or "shrub."

Spadefoots selected habitat with burrows created by other animals (e.g. gophers and ground squirrels), choosing sites without a pre-existing burrow with a 43 percent predicted probability (not significantly different from 50 percent), and choosing sites with a pre-existing burrow with 76 percent predicted probability (Table 3). Spadefoot selected burrows on flatter slopes with south-eastern aspects. For example, the model predicts that for a south-facing slope there would be a 2 percent chance of finding a spadefoot on a 30 degree slope, a 62 percent chance of finding a spadefoot on a 6 degree slope (the mean slope at our sites), and a 90 percent chance of finding a spadefoot on flat ground. For a 6-degree slope, a north-facing location would have a 49 percent probability of containing a spadefoot, a west-facing slope would have a 43 percent chance, and an east-facing slope would have a 78 percent chance (Table 3).

Soil Characteristics of Aestivation Locations

Total organic matter was not a statistically significant predictor of spadefoot presence. The soil-texture classification for all soil cores ranged from loamy sand to silt loam (Fig 4). The first principal component, indicating the overall soil composition across our sites, was not significant (Table 3). The second component, reflecting the degree to which sites varied from that overall soil composition, was significant. Spadefoots tended to prefer soils with less clay and more sand (Fig 4).

Fig 4. Ternary soil map showing percent composition of clay, sand, and silt at spadefoot sites.

On each side the arrow indicates which direction to read for that soil type (e.g. "clay" percentages are read horizontally). Black "X"s indicate the soil texture of pseudoabsence sites and black "O"s indicate soil texture of aestivation sites for our 15 spadefoots. The thick gray lines indicate principal component combinations of soil textures: principal component 1 (longer line) reflects the average soil texture of our sites, and principal component 2 (shorter line) reflects the deviation from that average. In color are model predictions using those two principal components: red signifies 99.7% probability of finding a spadefoot, while light yellow indicates 79.6% probability of finding a spadefoot.

Discussion

We documented details of the terrestrial activity of *S. hammondii*, which was previously unknown for the species [14,21]. No previous studies had been conducted on the upland habitat use of western spadefoots; the work done by Ruibal et al. [42] focused on *Spea multiplicata* when it was still thought to be a subspecies of *S. hammondii*. Our findings can establish

minimum buffer distances and type of habitat required for the conservation of the species and set a starting point for future study of this species' habitat needs – key information given that habitat loss is the driving factor in the western spadefoot being considered for listing under the Endangered Species Act [17] as well as its status as a Priority I Species of Special Concern in California [14].

Current conservation efforts for the western spadefoot include delineating buffer zones around known breeding pools. Based on our results, the 76 m buffer around vernal pools required by the California Department of Fish and Wildlife [13] encompasses 169/194 (87%) of spadefoot burrows. This amount may be enough habitat to maintain the local populations at our sites [12], but it does not consider habitat quality within the buffer area. The minimum terrestrial buffer distance of 368 m recommended by the U. S. Fish and Wildlife Service [15] would encompass all the spadefoot home ranges in our study. In rapidly urbanizing environments, implementation of a 368 m buffer around all breeding pools might not be feasible; therefore, important habitat elements such as aestivation locations and migration/dispersal corridors should be identified and conserved [27].

For the *S. hammondii* in coastal California, one important habitat element is grassland for burrowing, as indicated by our satellite-imagery based analysis. Other spadefoot species including *Spea intermontana* and the European spadefoot (*Pelobates fuscus),* were found to use grassland or areas with short vegetation rather than shrub habitat; however, both of those species were more likely to burrow in bare ground [43-44], unlike the *S. hammondii* at our sites which had over half of their burrows in areas characterized by duff. The duff present at both sites is the product of non-native annual grasses in the genera *Bromus* and *Avena* very little native grass was present at either site. The duff could act as cover for spadefoot movement and could also limit

evaporation, thus conserving soil moisture for spadefoot burrows [45]. In addition, three spadefoots aestivated within one meter of each other at the only tree-dominated habitat at one site. The tree's shade could keep the area cool during a hot summer, and it may also regulate soil moisture through leaf-litter mulch and hydraulic lift of the roots [46]. Tree cover could therefore be another important habitat element to conserve, and further study is warranted.

Another major habitat element may be the presence of mammal burrows. Like the sympatric California tiger salamander (*Ambystoma californiense*), the western spadefoot showed a preference for burrow placement adjacent to or in California ground squirrel (*Spermophilus beechyi*) and Botta's pocket gopher *(Thomomys bottae*) burrows [6]. The benefit of using mammal burrows include the ease of digging and the potential for optimal moisture conditions [47-48]. However, unlike salamanders, spadefoots are excellent diggers; therefore, we cannot assume that they were inside the gopher and ground squirrel burrows. In fact, during welfare checks, we found that one spadefoot had dug his own burrow adjacent to, but not in, a ground squirrel burrow. Use of pre-existing burrows does not come without risk; the spadefoots could be using the disturbed soil next to burrows to aid their digging without directly [illegible]ing the occupants of the burrows.

The spadefoots in our study stayed closer to the pool locations compared to sympatric species such as the Baja California treefrog (*Pseudacris hypochondriaca*), western toad (*Anaxyrus boreas*), and *A. californiense.* Brattstrom and Warren [49] found *P. hypochondriaca* 457–914 m from a lake in southern California, whereas *A. bore*[illegible]ave been found to move between an average of 218 m and 1105 m from breeding ponds depending on sex and site [50-51]. *A. californiense* migrates farther than all but one other salamander species with a median

distance of 556 m [52]. By contrast, the spadefoots in our study moved a mean maximum distance of 69 m (SD ± 61.48) and a maximum distance of 262 m from the pool. Considering that we found rain to be a significant predictor of spadefoot movement and distance moved, the ongoing drought during our study could have negatively impacted spadefoot movement distances. Rainfall was about 50% below the 60-year average during the 2012-2013 season [39]. In wetter years, the western spadefoot could potentially move much longer distances. For comparison, the closely related eastern spadefoot (*S. holbrookii*) can disperse an estimated maximum distance 449 m away from breeding pools, though this is under conditions with four times the amount of rain that fell in southern California during our study [24]. This comparison does indicate that we could expect much longer distances traveled in a year with more rainfall.

The movements we observed were not sufficient to connect the two sites studied to other known spadefoot breeding locations, the closest being a road rut 816 m from the Crystal Cove site. Knowledge of spadefoot dispersal is important for preserving the genetic diversity of *S. hammondii* populations [2]. Although we do not know if *S. hammondii* populations historically functioned as a metapopulation (i.e. with some exchange of individuals between subpopulations leading to increased genetic diversity and the recolonization of breeding pools following local extinction events [53]), our findings on spadefoot dispersal suggest that subpopulations may no longer be connected. Five years of drought dried pools and the subsequent lack of breeding heighten the likelihood of local extinction at both the Crystal Cove and the Laguna Coast Wilderness sites.

Because the spadefoot in our study utilized their terrestrial habitat differently from closely related and sympatric species, further research is warranted to determine if this difference was a result of low rainfall, or if it only applies to coastal populations. Western spadefoots are

found in a variety of habitats, including coastal sage scrub, chaparral, oak woodlands, grasslands, washes, and floodplains along the California coast through the Central Valley and into the Sierra Nevada foothills [14, 21]. Comparing movement and burrow preference of other populations or repeating the study in wetter years could provide additional insight on the natural history of this species while also informing land managers as to the terrestrial requirements of disparate spadefoot populations.

Acknowledgments

We thank our field volunteers and the Serrano Animal Hospital, in particular, Dr S. Weldy, Dr K. Krause and Dr N. Beaudet. This manuscript was improved by comments from D. Sandquist, J. Solera, and J. Carroll. Any use of trade names or specific product is for descriptive purposes only and does not imply endorsement of the U. S. Government. This study is contribution 502 of the U. S. Geological Survey Amphibian Research and Monitoring Initiative (ARMI).

References

1. Stuart SN, Chanson JS, Cox NA, Young BE, Rodrigues ASL, Fischman DL, et al. Status and trends of amphibian declines and extinctions worldwide. Science. 2004;306: 1783-1786.
2. Cushman SA. Effects of habitat loss and fragmentation on amphibians: A review and prospectus. Biol Conserv. 2006;128: 231-240.

3. Collins JP, Crump ML. Extinction in Our Times: Global Amphibian Decline. New York: Oxford University Press; 2009.
4. Hof C, Araujo MB, Jetz W, Rahbek C. Additive threats from pathogens, climate and land-use change for global amphibian diversity. Nature. 2011;480: 516-520.
5. Semlitsch RD, Skelly DK. Ecology and conservation of pool-breeding amphibians. In: Calhoun AJK, DeMaynadier PG, editors. Science and conservation of vernal pools in northeastern North America. Boca Raton: CRC Press, Taylor & Francis Group; 2007. pp. 128-42.
6. Trenham PC, Shaffer HB. Amphibian upland habitat use and its consequences for population viability. Ecol Appl. 2005;15: 115-1168.
7. Rittenhouse TAG, Semlitsch RD. Distribution of amphibians in terrestrial habitat and surrounding wetlands. Wetlands. 2007;27: 153-161.
8. McDonough C, Paton PWC. Salamander dispersal across a forested landscape fragmented by a golf course. J Wildl Manage. 2007;71: 1163-1169.
9. Goates MC, Hatch KA, Eggett DL. The need to ground truth 30.5m buffers: A case study of the boreal toad (*Bufo boreas*). Biol Conserv. 2007;138: 474-483.
10. Harper EB, Rittenhouse TAG, Semlitsch RD. Demographic consequences of terrestrial habitat loss for pool-breeding amphibians: Predicting extinction risks associated with inadequate size of buffer zones. Conserv Biol. 2008;22: 1205-1215.
11. Scott DE, Komoroski MJ, Croshaw DA, Dixon PM. Terrestrial distribution of pond-breeding salamanders around an isolated wetland. Ecology. 2013;94: 2537-2546.
12. Semlitsch RD, Brodie JR. Biological criteria for buffer zones around wetlands and riparian habitats for amphibians and reptiles. Conserv Biol. 2003;17: 1219-1228.

13. California Department of Fish and Wildlife. Appendix I: CDFW's conservation measures for biological resources that may be affected by program-level actions. In: Horizon Water and Environment, editors. San Joaquin River restoration program: Salmon conservation and research facility and related Management actions project-draft environmental impact report; 2013. pp. I1-I16.

14. Thomson RC, Wright AN, Shaffer HB. California amphibian and reptile species of special concern. Oakland: University of California Press; 2016.

15. U. S. Fish and Wildlife Service. Recovery plan for vernal pool ecosystems of California and southern Oregon. Portland (OR): U. S. Fish and Wildlife Service; 2005. pp. II-220-II-235.

16. Stebbins RC, McGinnis SM. Field guide to amphibians and reptiles of California. California Natural History Guides. Revised Edition. Berkeley: University of California Press; 2012.

17. U. S. Fish and Wildlife Service. Evaluation of a petition to list the western spadefoot toad as an endangered or threatened species under the Act. Federal Register. 2015;80: 37578-37579.

18. Fisher RN, Shaffer HB. The decline of amphibians in California's Great Central Valley. Conserv Biol. 1996;10: 1387-1397.

19. Dimmitt MA, Ruibal R. Environmental correlates of emergence in spadefoot toads (*Scaphiopus*). J Herpetol. 1980;14: 21-29.

20. Keeley JE, Zedler PH. Characterization and global distribution of vernal pools. In: Witham CW, Bauder ET, Belk D, Ferren WR, Jr., Ornduff R, editors. Ecology,

conservation, and management of vernal pool ecosystems. Proceedings from a 1996 Conference. California Native Plant Society, CA; 1998. Pp.1-14.

21. Morey SR. *Spea hammondii*. In: Lannoo MJ, editor. Amphibian declines: The conservation status of United States species. Berkeley: University of California Press; 2005. pp. 514-517.

22. Beaupre SJ, Jacobsen ER, Lillywhite HB, Zamudia K. Guidelines for use of live amphibians and reptiles in field and laboratory research. Herpetological Animal Care and Use Committee (HACC) of the American Society of Ichthyologists and Herpetologist, 2004 Second Edition.

23. NOAA. 2016. Daily summary details for Laguna Beach, CA. Available from: http://www.ncdc.noaa.gov/cdoweb/datasets/GHCND/stations/GHCND:USC00044647/detail. Cited 1 October 2016.

24. Timm BC, McGarigal K, Cook RP. Upland movement patterns and habitat selection of adult eastern spadefoots (*Scaphiopus holbrookii*) at Cape Cod National Seashore. J Herpetol. 2014;48: 84-97.

25. Hoover MD, Lunt HA. A key for the classification of forest humus types. Soil Science Society Proceedings. 1952;1952: 368-370.

26. Beyer HL. Geospatial modelling environment, Version 0.7.2.1 [software]. 2012 [cited 2015 Sep 20]. Available from: http://www.spatialecology.com/gme.

27. Baldwin RF, Calhoun AJK, deMaynadier PG. Conservation planning for amphibian species with complex habitat requirements: a case study using movements and habitat selection of the wood frog *Rana sylvatica*. J Herpetol. 2006;40: 442-453.

28. Kirby ME, Lund SP, Patterson WP, Anderson MA, Bird BW, Ivanovici L, et al. A Holocene record of Pacific decadal oscillation (PDO)-related hydrologic variability in southern California (Lake Elsinore, CA). J Paleolimnol. 2010;44: 819-839.

29. Dean WE, Jr. Determination of carbonate and organic matter in calcareous sediments and sedimentary rocks by loss on ignition: comparison with other methods. J Sediment Petrol. 1974;44: 242-248.

30. Wentworth CK. A scale of grade and class terms for clastic sediments. J Geol. 1922;30: 377-392.

31. Sheldrick BH, Wang C. Particle size distribution. In Carter MR, editor. Soil sampling and methods of analysis. Boca Raton: Lewis Publishers; 1993. pp. 499-511.

32. Getz WM., Wilmers CC. A local nearest-neighbor convex-hull construction of home ranges and utilization distributions. Ecography. 2004;27: 489-505.

33. Getz WM, Fortmann-Roe S, Cross PC, Lyons AJ, Ryan SJ, Wilmers CC. LoCoH: Nonparametric kernel methods for constructing home ranges and utilization distributions. PLoS ONE. 2007: 2(2): e207. doi:10.1371/journal.pone.0000207.

34. Signer J, Balkenhol N. Reproducible home ranges (rhr): a new, user-friendly R package for analysis of wildlife telemetry data. Wildl Soc Bull. 2015. doi:10.1002/wsb.539.

35. R Core Team. R: A language and environment for statistical computing. R Foundation for Statistical Computing, Vienna, Austria. 2014. Available from: http://www.R-project.org/.

36. Manly BFJ, McDonald LL, Thomas DL, McDonald TL, Erickson WP. Resource selection by animals: Statistical design and analysis for field studies. 2nd ed. Dordrecht: Springer; 2002.

37. Fournier DA, Skaug HJ, Ancheta J, Ianelli J, Magnusson A, Maunder M, et al. AD model builder: Using automatic differentiation for statistical inference of highly parameterized complex nonlinear models. Optim Methods Softw. 2012;27: 233-249.

38. Zuur AF, Ieno EN, Walker NJ, Saveliev AA, Smith GM. Mixed effects models and extensions in ecology with R. New York: Springer; 2009.

39. Orange County Public Works. 2013. OC watersheds Laguna Beach rain station number 100, Lat. 33-32-49, Long. -117-46-53. Available from: http://ocwatersheds.com/rainrecords/rainfalldata/historic_data/ rainfall_data. Cited 20 September 2015.

40. University of Texas McDonald Observatory. StarDate: Moon phases. 2013. Available from: http://stardate.org/nightsky/moon. Cited 20 September 2015.

41. Gray MJ, Miller DL, Smith LM. Coelomic response and signal range of implant transmitters in *Bufo cognatus*. Herpetol Rev. 2005;36: 285-288.

42. Ruibal R, Tevis Jr. L, Roig V. The terrestrial ecology of the spadefoot toad *Scaphiopus hammondii*. Copeia. 1969;1969: 571-584.

43. Eggert C, Guyetant R. Safeguard of a spadefoot toad (*Pelobates fuscus*) population: a French experience. In: Ferri V, editor. Atti del Terza Convergno Salvaguardia Anfibi. Progetto ROSPI & Museo cantonale di Storia Naturale di Lugano. Penne: Cogecstre Ediz; 2002. pp. 47-52.

44. Garner JL. Movement and habitat-use of the great basin spadefoot (*Spea intermontana*) at its northern range limit. M.S. Thesis, Thompson Rivers University, Kamloops, British Columbia, Canada. 2012.

45. D’Antonio CM, Malmstrom C, Reynolds SA, Gerlach J. Ecology of invasive non-native species in California grassland. In: Stromberg MR, Corbin JD, D’Antonio CM, editors. California grasslands: Ecology and management. Berkeley: University of California Press; 2007. pp. 67-83.

46. Vetaas OR. Micro-site effects of trees and shrubs in dry savannas. J Veg Sci. 1992;3: 337-344.

47. Laundre, JW. Effects of small mammal burrows on water infiltration in a cool desert environment. Oecologia. 1993;94: 43-48.

48. Reichman OJ, Seabloom EW. The role of pocket gophers as subterranean ecosystem engineers. Trends Ecol and Evol. 2002;17: 44-49.

49. Brattstrom BH., Warren JW. Observations on the ecology and behavior of the Pacific treefrog, *Hyla regilla*. Copeia. 1955;1955: 181-191.

50. Muths E. Home range and movements of boreal toads in undisturbed habitat. Copeia. 2003;2003: 160-165.

51. Bartelt PE, Peterson CR, Klaver RW. Sexual differences in the post-breeding movements and habitats selected by western toads (*Bufo boreas*) in southeastern Idaho. Herpetologica. 2004;60: 455-467.

52. Searcy CA, Gabbai-Saldate E, Shaffer HB. Microhabitat use and migration distance of an endangered grassland amphibian. Biol Conserv. 2013;158: 80-87.

53. Smith MA, Green DM. Dispersal and the metapopulation paradigm in amphibian ecology and conservation: are all amphibian populations metapopulations? Ecography. 2005;28: 110-128.

Supporting Information

S1 Table. Spadefoot summary data. Table giving the length, mass, sex, capture date, number of telemetry fixes, number of burrows used, maximum distance from pool in meters, mean distance between burrows in meters, standard deviation of distance between burrows, minimum convex polygon home range in square meters, percent grass in that home range, depth of burrow in centimeters, and site location of each animal in our study.

Figure1

Click here to download Figure Fig1.tiff

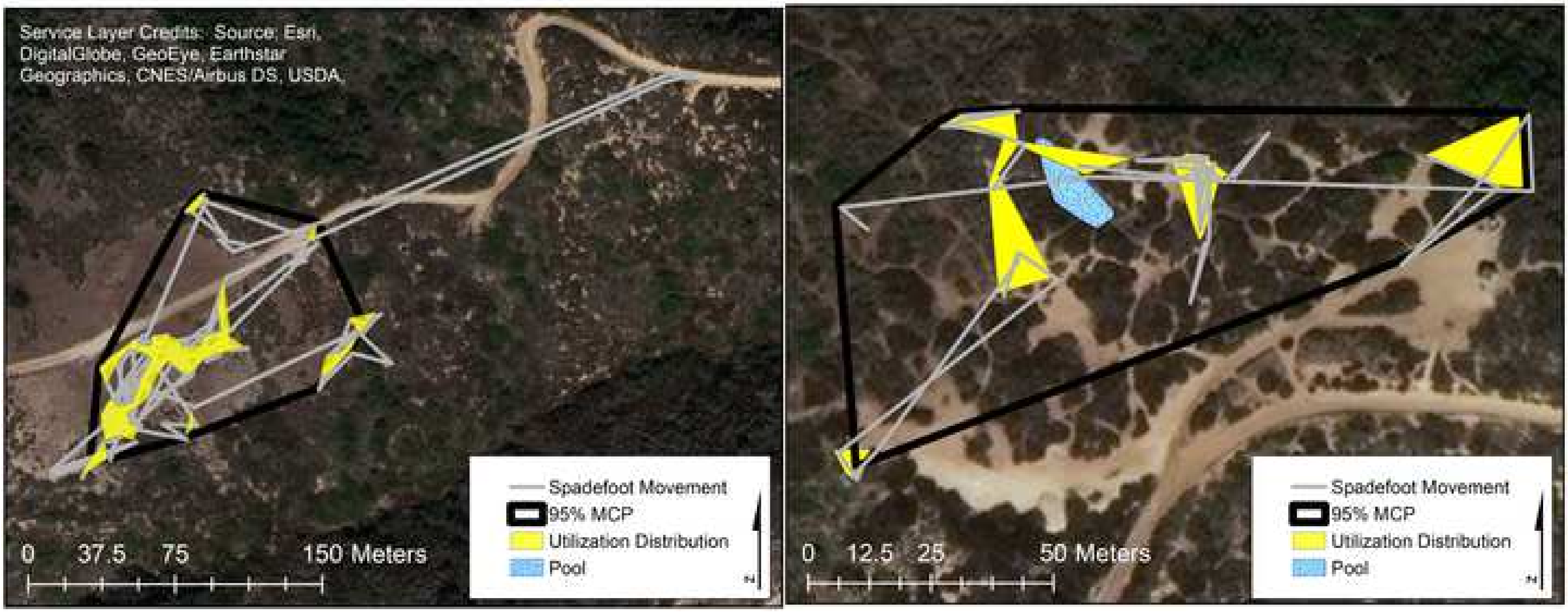

Figure2

Click here to download Figure Fig2.tiff

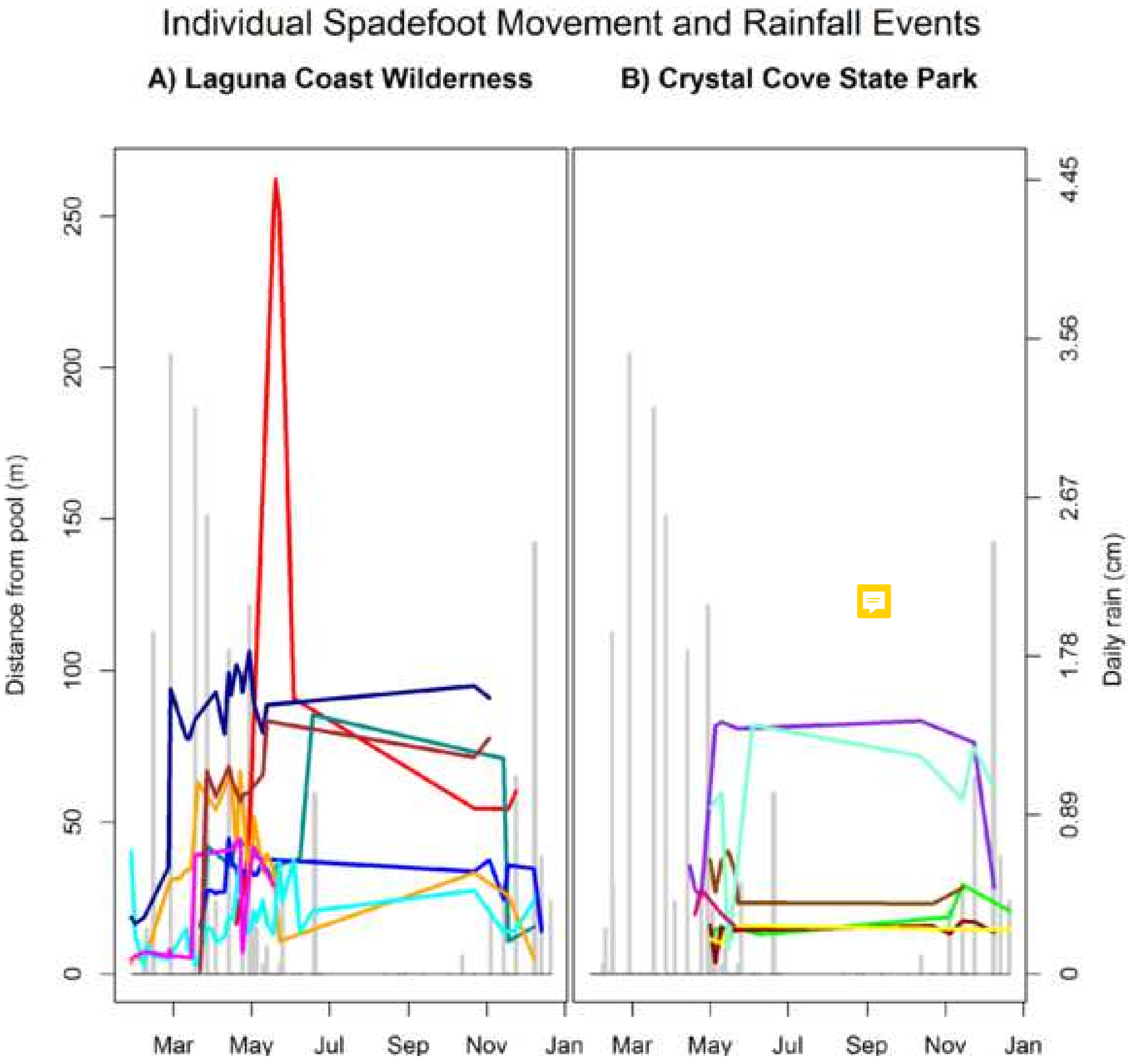

Figure3

Click here to download Figure Fig3.tiff

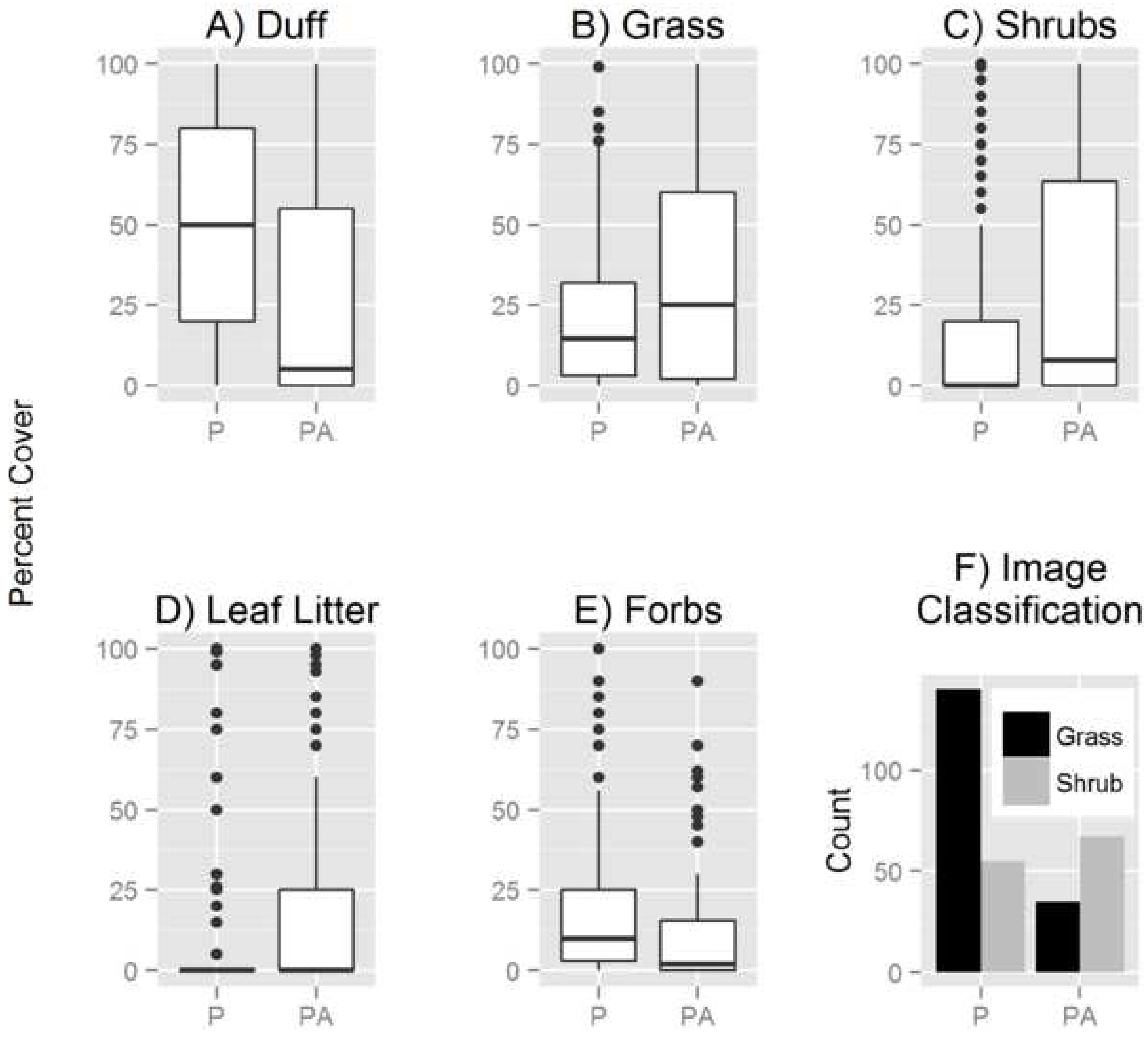

Figure4

Click here to download Figure Fig4.tiff

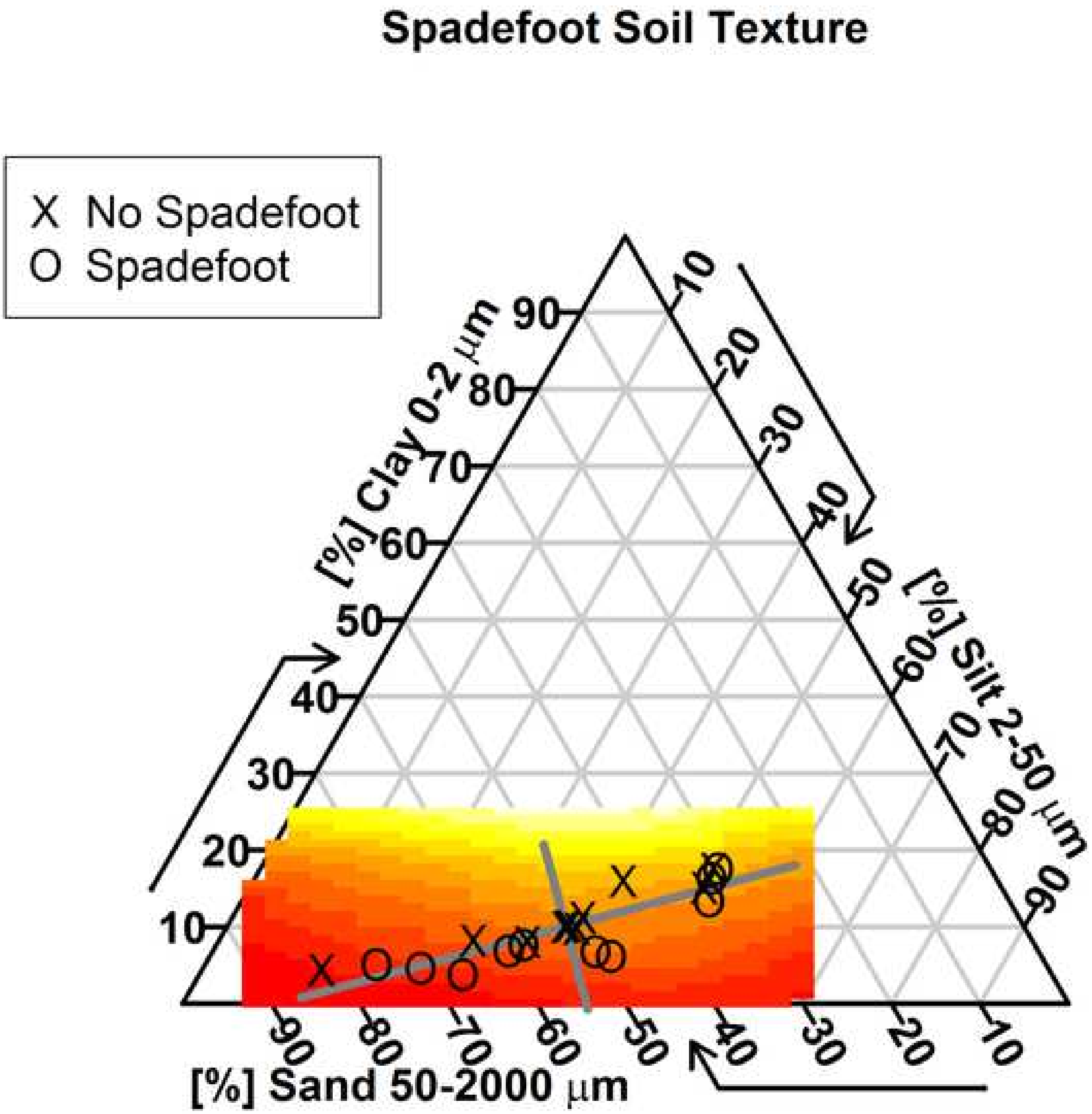

Click here to access/download
Supporting Information
S1_Table.xls

Click here to access/download
Data Review URL
https://datadryad.org/review?doi=doi:10.5061/dryad.835
9820
